# Role of research Laboratories in pandemic and epidemic response in the Eastern Mediterranean Region: Experiences from COVID‐19, avian influenza, and MERS‐CoV

**DOI:** 10.1111/irv.13257

**Published:** 2024-02-11

**Authors:** Mona Mahmoud, Rebecca Badra, Ahmed Kandeil, Rabeh El‐Shesheny, Jad Abdallah, Mohamed A. Ali, Ghazi Kayali

**Affiliations:** ^1^ Human Link DMCC Dubai United Arab Emirates; ^2^ Center of Scientific Excellence for Influenza Virus Institute of Environmental Research and Climate Changes, National Research Centre Giza Egypt; ^3^ Multi‐Omics Laboratory, School of Pharmacy Lebanese American University Byblos Lebanon

**Keywords:** Eastern Mediterranean Region, epidemic, pandemic, preparedness, research laboratories, response

## Abstract

We share the experience of research laboratories in the Eastern Mediterranean Region (EMR) that contributed to preparedness and response to highly pathogenic avian influenza (HPAI), Middle‐East respiratory syndrome coronavirus (MERS‐CoV), and coronavirus disease (COVID‐19). Research groups in the region were pivotal in identifying, characterizing the pathogens and describing their evolution, distribution, transmission routes, and the immunological profile of exposed populations. They demonstrated the capacity to develop and test antivirals and potential vaccines. The EMR experience is a model of how national systems can work with researchers to improve regional preparedness and response to future epidemics and pandemics.

## 1 INTRODUCTION

**FIGURE 1:**
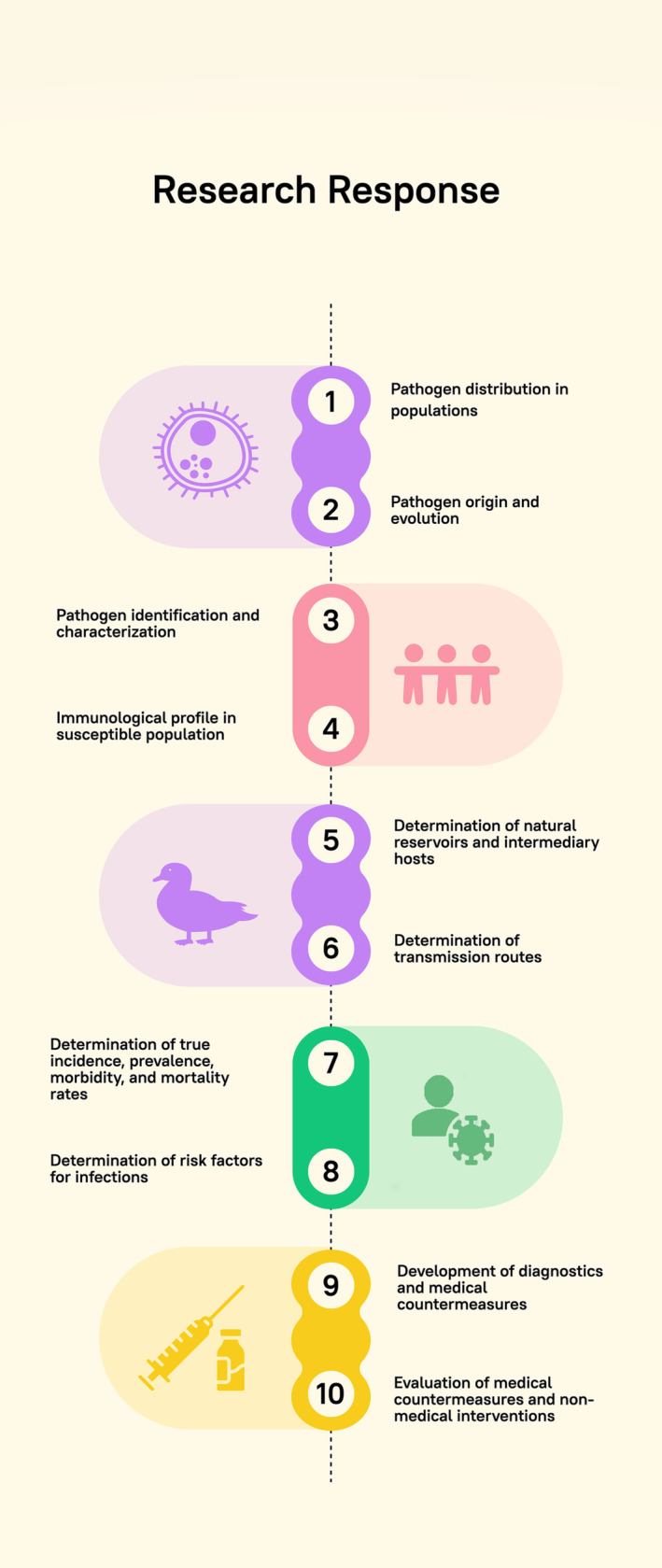
Potential inputs from research institutions for pandemic and epidemic preparedness and response.

Emerging and reemerging infectious diseases remain a major concern in the Eastern Mediterranean region (EMR). Numerous factors, including the expansion of human–animal interfaces, accelerated urbanization, and humanitarian emergencies that weaken healthcare and public health systems contribute to the emergence and rapid spread of epidemics and pandemics in the region. Prevention, detection, and response capacities are key to adequate pandemic and epidemic preparedness. The EMR continues to report gaps in preparedness even though improvements have occurred following the emergence of highly pathogenic avian influenza (HPAI) H5N1 in 2005, the 2009 influenza pandemic, the emergence of the MERS‐CoV epidemic in 2012, and more recently, the COVID‐19 pandemic. Although national and regional public and animal health systems play a lead role in response through diagnosis and detection, research laboratories such as academic laboratories, government laboratories, and private sector laboratories that are engaged in basic and applied research play a pivotal role in prevention, detection, and response. Specifically, research can identify and characterize the pathogen of interest and describe its origin, evolution, natural reservoirs, and intermediary hosts. In exposed populations, research can provide information on the distribution of the pathogen in the population, the transmission routes, and the immunological profile of the population. Research can also lead to designing proper diagnostics and medical countermeasures. Figure 1 illustrates potential inputs from research laboratories for respiratory viruses' pandemic and epidemic preparedness and response. We share the experience of research laboratories in the EMR that contributed to preparedness and response to HPAI, MERS‐CoV, and COVID‐19.

HPAI H5N1 was first introduced to the EMR in Turkey in 2005^1^ and into Egyptian poultry in 2006.^2^ Low pathogenic avian influenza (LPAI) H9N2 virus was detected in 2011. Both viruses became endemic in the domestic poultry population. Consequently, Egypt reported the highest number of (HPAI) H5N1 human cases and reported few cases of (LPAI) H9N2.^2^ In response, a research surveillance program for avian influenza viruses was established.^3^ This program provided insights into the epizootic situation showing that H5N1 and H9N2 viruses were constantly evolving genetically and antigenically and that both viruses were co‐circulating and co‐infecting.^3^ Consequently, phylogenetic analysis demonstrated that avian influenza H9N2 viruses in Egypt were reassorting with other avian influenza viruses to produce antigenically distinct species.^4^ This program further detected the introduction of H5NX clade 2.3.4.4b viruses that have been spreading extensively among wild birds and poultry globally.^5^ Those scientific findings assisted animal health authorities in filling gaps in their surveillance activities.

Importantly, this program was conducted at the human‐animal interface, focusing on humans exposed to poultry. Cohort studies measured the incidence and seroprevalence of avian influenza viruses in poultry growers highlighting that national surveillance systems were underestimating the true disease burden.^6^ Additionally, as Egypt focused on vaccinating poultry against H5 and H9 viruses, this program allowed studying vaccine efficacy and produced seed vaccines better suited for use in Egypt, thus assisting animal health authorities in responding to the epizootic with proper medical countermeasures.^7^

The first case of MERS‐CoV infection was reported in June 2012 in the Kingdom of Saudi Arabia.^8^ Since then, several cases and clusters of infections have been documented. In response, researchers determined that camels are a natural reservoir for the virus, enabling public health systems to attempt to reduce camel‐to‐human transmission.^9^ In early 2014, the full genome sequence of MERS‐CoV was obtained from a nasal swab sample from a dromedary camel from Egypt, showing that the viruses infecting dromedaries were genetically extremely close to MERS‐CoV infecting the human population. This conclusion was solidified by a work from Saudi Arabia obtaining identical MERS genomes from a human and the camel he was exposed to.^10^ Surveillance in dromedaries in several countries in the region, including Egypt, Tunisia, Iraq, Saudi Arabia, and Senegal, showed that MERS commonly infects camels and may spill over to other livestock in contact with camels.^11^ Phylogenetic analysis suggested that the sequences from Egypt are of clade C and clustered together with the MERS‐CoV detected in Africa.^12^ Together, those research findings were critical in understanding the reservoir, potential transmission routes, viral characteristics, and viral dynamics within animal populations. This is critical for multisectoral response to a zoonotic disease.

The World Health Organization (WHO) declared a Public Health Emergency of International Concern (PHEIC) on January 30, 2020 and characterized COVID‐19 outbreak as a pandemic on March 11, 2020.^13^ Approximately 5 million cases, including 121,778 deaths, were documented in the EMR by the end of 2020. Research laboratories in several EMR countries provided important scientific findings with potential policy implications. For instance, between February 2020 and January 2022, 1230 SARS‐CoV‐2 sequences from Lebanon were analyzed. Three variants of concern, including alpha, delta, and omicron, were detected within the analyzed strains. Additionally, mutations in the structural, non‐structural, and accessory proteins were detected by the mutational survey.^14^ A similar study was conducted in Egypt where full genomes of 1256 SARS‐CoV‐2 strains were analyzed between March 2020 and May 2021. Findings suggested that one emerging lineage of the Egyptian SARS‐CoV‐2 had similar mutations as the variants of concern.^15^ Such information is important for public health authorities to understand the characteristics of circulating viruses and thus plan interventions accordingly.

A cross‐sectional serological study was conducted between October 2020 and April 2021 to assess the prevalence of neutralizing antibodies against SARS‐CoV‐2 and determine accompanying factors in Lebanon. The study revealed that 58.95% of the study population showed neutralizing antibodies. Individuals who had to leave for work during the pandemic and lived in suburban areas that did not adhere to preventive measures suffered from higher positivity rates.^16^ Another seroprevalence study was conducted in Egypt. It showed that 30% of the study population had antibodies against SARS‐CoV‐2.^17^ Both studies were conducted before the vaccine rollout, providing public health authorities with an estimate of the true spread of COVID‐19 in their populations. Furthermore, research teams teamed with public health experts to study whether plasma from COVID‐19 patients can be used as a treatment for severe cases, an example of research's role in studying potential countermeasures.^18^

An essential approach to defend against SARS‐CoV‐2 is vaccination. Research groups in the region developed and tested potential vaccines. In Egypt, an inactivated SARS‐CoV‐2 vaccine was developed and proved safe and efficacious in laboratory animals.^19^ Upon rollout of WHO‐approved vaccines, such as ChAdOx1 nCov‐19 (Oxford/AstraZeneca), BNT162b2 (Pfizer/BioNTech), BBIBP‐CorV (Sinopharm), and Ad26.COV2.S COVID‐19 (Janssen/Johnson), a serological study was conducted to examine antibodies induced by different vaccines against the frequent variants of SARS‐CoV‐2. Findings concluded that adenovirus vector‐based vaccines (Oxford/AstraZeneca) and (Janssen/Johnson) and the mRNA vaccine (Pfizer/BioNTech) were effective against the majority of common strains of SARS‐CoV‐2, while BBIBP‐CorV inactivated vaccine (Sinopharm) was less efficient.^20^ Such information can assist public health authorities in adjusting their vaccination campaigns using the most effective vaccines. Additionally, research laboratories tested various compounds for their antiviral activity, thus supporting case management decisions.

This short communication illustrated the crucial role that research laboratories performed in guiding preparedness plans and assisting during response. Though research in the EMR is not as advanced as in high‐income countries, several research groups in several countries have been active and prolific in studying various aspects of high‐consequence pathogens. Nonetheless, the role of those research entities has been, for the most part, isolated from public and animal health policies. At the national and regional levels, it is essential to start working on bridging this gap between research and policy by involving relevant stakeholders such as human and animal health authorities, universities, and international organizations under a One Health knowledge translation and management committee. This committee would be responsible for elucidating the process of communication between researchers and policy‐makers to build trusted relationships, proposing adaptable strategies and policies from scientific evidence, and providing guidance to decision‐makers to enhance emergency preparedness and management.

Toward this, several steps are suggested:

1. Conduct mapping exercises to understand the research capabilities nationally and regionally.
2. Involve research in risk assessment exercises.
3. Develop national and regional research agendas.
4. Conduct joint research projects aimed at answering scientific questions of national and regional importance.
5. Develop mechanisms to translate research findings into actionable public and animal health policies.

The COVID‐19 pandemic provided important lessons on why policies and interventions must be evidence‐based. Such evidence is produced through scientific research conduits and then carefully interpreted and incorporated into policy. The EMR played a leading role in demonstrating how national systems can work together with researchers to improve the region's capacities to prevent, detect, and respond to future epidemics and pandemics. As the threats of avian influenza, MERS‐CoV, and SARS‐CoV‐2 continue, such collaborations continue to be beneficial and require further strengthening.

## AUTHOR CONTRIBUTIONS

**Mona Mahmoud**: Data curation; formal analysis; investigation; writing—original draft preparation; writing—review and editing. **Rebecca Badra:** Methodology; writing—original draft preparation; writing—review and editing. **Ahmed Kandeil:** Data curation; formal analysis; resources. **Rabeh El‐Shesheny:** Data curation; formal analysis; resources**. Jad Abdallah**: Resources; writing—review and editing. **Mohamed A. Ali:** Supervision**. Ghazi Kayali:** Conceptualization; methodology; supervision; validation; visualization; writing—review and editing.

## CONFLICT OF INTEREST STATEMENT

The authors declare no competing interests.

## Data Availability

Data sharing is not applicable to this article as no new data were created or analyzed in this study.
